# Large Variability in Response to Future Climate and Land-Use Changes of François’ Langur in China

**DOI:** 10.3390/biology15070526

**Published:** 2026-03-26

**Authors:** Qixian Zou, Bingnan Dong, Fan Zhang, Siyao Li, Xing Fan, Jialiang Han

**Affiliations:** 1 Guizhou Mayanghe National Nature Reserve Administration, Tongren 565300, China; 2 Sichuan Academy of Forestry, Chengdu 610000, China; 3 Chengdu Botanical Garden, Chengdu 610000, China; 4 Office of Academic Afairs, Chengdu University, Chengdu 610000, China

**Keywords:** François’ langur, climate change, land use change, current distributions, future distributions

## Abstract

Understanding how climate and land-use change influence habitat suitability is essential for the conservation of the François’ langur (*Trachypithecus francoisi*). In this study, climatic, land-use, and topographic variables were integrated to model the current distribution and future dynamics of suitable *T. francoisi* habitats in southwestern China. The model performed well, climatic factors were the primary determinants of distribution, particularly precipitation of the driest month (BIO14), mean diurnal temperature range (BIO2), and precipitation seasonality (BIO15); additionally, forest cover, slope, and elevation further improved model performance. Suitable habitat currently covers 53,109 km^2^ (10.75% of the study area) and is mainly concentrated in Chongqing and Guizhou, with smaller areas in Guangxi. Future projections indicate substantial habitat redistribution and an overall decline in suitable area under both scenarios. By the 2050s and 2070s, suitable habitats will show strong spatial turnover, with coexistence of retained, newly suitable, and lost areas. Suitable habitat is projected to shift toward northern areas. These results suggest that conservation priorities should shift focus northward under climate warming, with emphasis on protecting mountainous refuges and improving habitat connectivity.

## Simple Summary

François’ langur is a folivorous primate, primarily inhabiting karst limestone forests. This study evaluated how climate and land-use change may reshape habitat suitability across southwestern China. Quantitative modeling indicates that currently suitable habitat covers approximately 53,109 km^2^ (10.75% of the study area), with the most continuous patches concentrated in Chongqing and Guizhou. Habitats in Guangxi have experienced notable declines, mirroring regional population reductions. Under future scenarios, the total suitable area is projected to undergo substantial spatial turnover and an overall reduction. These habitats are expected to shift toward northern and higher-elevation regions as the species tracks suitable microclimates. The results suggest that mountainous regions in Chongqing and northern Guizhou will likely serve as critical future refugia. These findings emphasize the need to adjust conservation priorities toward northern refugial landscapes and improve habitat connectivity to sustain future populations.

## 1. Introduction

Climatic oscillations have historically played a pivotal role in shaping global biodiversity by driving species distributions, population isolations, and secondary gene flow [1,2,3]. Global biodiversity patterns are increasingly driven by two major forces: climate change and land-use change [3,4]. Climate change alters temperature and precipitation regimes, modifying the environmental suitability of future habitats. Land-use change reflects human activities that reshape the Earth’s surface, modifying habitat structure, availability, and spatial configuration. These processes are closely related to environmental change and ecological quality [3,4]. They strongly influence species distributions, dispersal, and long-term persistence. Climate change and land-use change also profoundly affect animal phenology, behavior, and physiology, and these multifaceted global changes pose severe threats to global biodiversity [4,5].

Many studies have assessed the independent effects of climate change or land-use change on species’ range shifts, but results have varied [6,7,8]. Climate change affects broad-scale habitat suitability, whereas land-use change drives localized habitat loss and fragmentation, impairing landscape connectivity. Emerging evidence suggests that these stressors do not operate in isolation, and they interact to jointly determine the availability and accessibility of stable refugia [9,10]. Their combined effects may either amplify or buffer the impact of a single driver, with species-specific traits often mediating the direction and magnitude of these responses [11,12,13]. Identifying taxa vulnerable to these dual pressures and refining predictive models of range shifts under integrated scenarios are critical for modern biodiversity conservation [14,15]. Among the world’s 522 primate species, about 68% are currently at risk of extinction [16,17,18,19,20]. Suitable habitats for primates in China are projected to continue declining and become increasingly restricted to smaller, fragmented areas due to the compounding pressures of climatic shifts and human encroachment [16,21]. Over approximately the past 2000 years, rising temperatures in the Northern Hemisphere have led to a northward contraction in the distribution of snub-nosed monkeys (*Rhinopithecus*) [22,23]. A similar historical decline is observed in other primates, such as the recently described extinct gibbon Nomascus imperialis (*formerly Junzi imperialis*), which inhabited central China during the late Holocene before its extinction [24].

François’ langur (*Trachypithecus francoisi*), classified as ‘Endangered’ by the International Union for Conservation of Nature, holds the status of a first-grade protected wildlife species in China [25]. Recent research findings suggest that the total population of François’ langur is estimated to be approximately 2000 individuals, distributed across Vietnam and China [25,26]. Vietnam is home to an estimated 160–190 individuals, while China harbors a more substantial population of around 1500 individuals [27,28,29]. The preference of François’ langur for primary and secondary forests, coupled with a reliance on specific plant species for sustenance, underscores the species’ ecological specialization. The very habitats crucial to its survival are increasingly imperiled by human-induced factors, notably deforestation, logging, and agricultural expansion [30,31]. The profound implications of habitat loss extend beyond the immediate displacement of the François’ langur, permeating through intricate ecological networks. Fragmentation of the habitat of François’ langur disrupt established corridors for movement and compromises gene flow among populations, exacerbating the species’ vulnerability [29,30]. The survival of François’ langur has become emblematic of the broader challenges confronting biodiversity conservation in the face of anthropogenic pressures.

Based on distribution records of François’ langur in China from 2010 to 2024, we developed Biomod2 models to predict potential habitat suitability under current and future climate and land-use scenarios (2050s and 2070s). The current study aims to address the following questions: (*i*) What are the spatial distribution characteristics of suitable habitats for François’ langur under shifting climate and land-use patterns, and what are the primary environmental drivers? (*ii*) Where are the primary conservation gaps located? The findings will provide a quantitative basis for the field monitoring, habitat selection, and protected area planning for François’ langur.

## 2. Materials and Methods

### 2.1. Study Area and Species Occurrence Data

**Figure 1:**
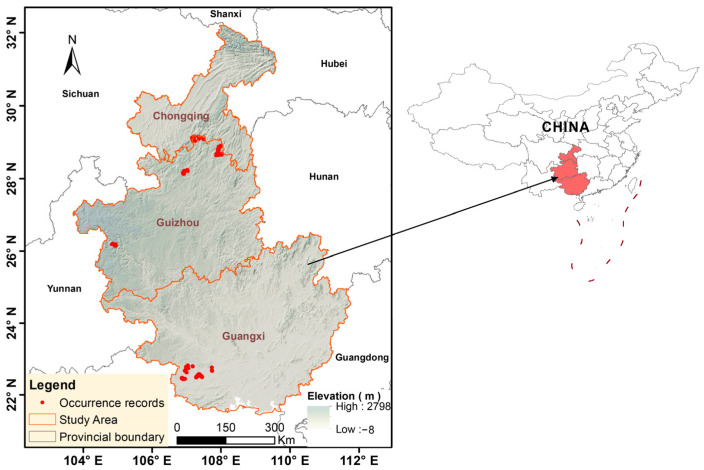
Location of study area and the distribution of François’ langur.

The occurrence records for François’ langur were compiled from two primary sources. First, we conducted a comprehensive review of the published literature spanning 2010 to 2024 [27,31,32,33,34]. A total of 316 raw occurrence points were gathered from the species’ distribution ranges across Guizhou, Chongqing, and Guangxi provinces. Second, supplementary field investigations were carried out between 2015 and 2024 at all known population sites in Guizhou and Chongqing; during these surveys, species presence was documented through direct visual observations and the identification of fecal remains (Figure 1).

### 2.2. Data Filtering and Spatial Thinning

To minimize spatial autocorrelation and prevent model overfitting, we applied a spatial thinning procedure to the occurrence data. We used the ‘sp Thin’ package in R 3.3 to filter records [35], ensuring a minimum distance of 1 km between any two points, which corresponds to the 1 km × 1 km spatial resolution of our bioclimatic variables. After removing duplicate and spatially clustered records, 218 high-quality occurrence points were retained for the final Biomod2 modeling and subsequent analysis (Figure 1).

### 2.3. Topographic, Climate and Land-Use Variables

#### 2.3.1. Topographic Variables

Four topographical variables were selected for the Biomod2 model: elevation, slope, aspect, and distance to rivers. The elevation, slope, and aspect layers were derived from a Digital Elevation Model (DEM) with a spatial resolution of 1 km × 1 km. This dataset was obtained from the Geospatial Data Cloud (http://www.gscloud.cn), hosted by the Computer Network Information Center of the Chinese Academy of Sciences. The slope and aspect were processed using the Surface Analyst Tool in ArcGIS 10.5. The Euclidean Distance Tool was employed to generate the layer representing the distance to the nearest river based on hydrological data.

#### 2.3.2. Current and Future Climate

Current bioclimatic data were obtained from the China Meteorological Forcing Dataset [12,36]. This dataset provides monthly gridded temperature and precipitation measures with a spatial resolution of 1 km × 1 km. Given that our species occurrence records were collected primarily between 2010 and 2020, we extracted the monthly data for this period and calculated 19 bioclimatic variables using the biovars function in the R 3.3 package [12]. We obtained the same bioclimatic variables from the World Clim 2.1 Database [37] for the 2050s (2041–2060) and 2070s (2061–2080). We selected two Shared Socio-economic Pathways (SSPs)—SSP1-2.6 (optimistic) and SSP5-8.5 (pessimistic)—based on an ensemble of six global circulation models (GCMs) from CMIP6: BCC-CSM2-MR, CNRM-CM6-1, CNRM-ESM2-1, IPSL-CM6A-LR, MIROC6, and MRI-ESM2-0. These GCMs were chosen for their high consensus and predictive performance in the East Asian region [12,38].

#### 2.3.3. Land-Use Variables

Current and future land-use data were sourced from the FROM-GLC datasets at a 1 km resolution [39]. This dataset simulates global land-use dynamics (including ten categories such as forest, cropland, and shrubland) under scenarios that correspond to the SSP framework. For the current period, we utilized land-use data from the year nearly coincident with our field surveys to reflect the habitat status at the time of observation. Preliminary analysis confirmed that François’ langur occurrences were primarily associated with high forest cover and low cropland proportion, consistent with the species’ known habitat requirements. For future projections, we extracted land-use variables for the 2050s and 2070s under the same SSP scenarios (SSP1-2.6 and SSP5-8.5).

To mitigate multicollinearity, we employed the ‘usdm’ package in R, using the ‘vifcor’ and ‘vifstep’ functions [12]. Variables were selected based on a Pearson correlation coefficient (|r| < 0.7) and a Variance Inflation Factor (VIF < 5) [12]. Five bioclimatic and three land-use variables were retained for MaxEnt modeling. The climate variables included: Mean of Monthly Temperature (max temp-min temp) (BIO2), Temperature Seasonality (BIO4), Max Temperature of Warmest Month (BIO5), Precipitation of Driest Month (BIO14), and Precipitation Seasonality (BIO15). The land-use variables included the proportions of cropland, forest, shrubland.

#### 2.3.4. Model Processing

Species distribution models were developed using an ensemble framework. This framework was implemented in the R package Biomod2 to improve predictive robustness [40]. Three algorithms with strong performance in ecological niche modeling were included: Maximum Entropy (MaxEnt), Random Forest (RF), Generalized Boosting Model (GBM), Generalized Linear Model (GLM), and Multivariate Adaptive Regression Splines (MARS). Specifically, MaxEnt was implemented using linear, quadratic, and product features to handle presence-only data. RF and GBM were utilized to capture complex nonlinear responses and high-order interactions through ensemble tree-based learning. To maintain interpretability, the GLM was fitted with linear and quadratic terms, while MARS was used to detect non-linear relationships and environmental thresholds through piecewise linear regressions [41,42]. Because the GBM and GLM require absence data, pseudo-absence points equal to the number of occurrence records were randomly generated across the study area, while 10,000 background points were sampled for MaxEnt [12]. Models were run ten times using five-fold cross-validation, with 80% of the data used for calibration and 20% for evaluation. Model performance was assessed using the Area Under the Curve (AUC) and True Skill Statistic (TSS), and only models with AUC > 0.8 and TSS > 0.6 were retained [41]. Final ensemble predictions were produced by combining selected models using the median of replicate runs, reducing uncertainty associated with individual algorithms.

Variable importance was evaluated using a permutation approach that quantifies the change in correlation between predictions and observations after randomly shuffling each predictor [43]. The calibrated ensemble models were projected onto baseline environmental conditions and future scenarios representing climate change alone and combined climate–land-use changes under different time periods and SSPs. Continuous suitability outputs were converted into binary presence–absence maps using the optimal TSS threshold. To account for uncertainty among climate projections, results from multiple Global Climate Models were averaged to generate consensus predictions of future species distributions [40,44,45].

#### 2.3.5. Changes in the Spatial Pattern of the Suitable Area for François’ Langur

Spatial units with predicted occurrence probabilities ≥ 0.35 were classified as suitable habitat, whereas those with probabilities < 0.35 were considered unsuitable. Based on this threshold, binary presence–absence (1/0) maps were generated for the current period and for future scenarios (SSP1-2.6 and SSP5-8.5) for the 2050s and 2070s, with suitable areas assigned a value of 1 and unsuitable areas assigned a value of 0. Using these binary matrices, changes in the spatial pattern of suitable habitat were quantified by comparing each future scenario with the current distribution. Habitat change was categorized into four types: expansion areas (0 → 1), contraction areas (1 → 0), unchanged areas (1 → 1), and continuously unsuitable areas (0 → 0). These transitions were used to calculate and compare the extent and spatial dynamics of suitable habitat under the different future climate scenarios.

## 3. Results

### 3.1. Model Performance and Variable Importance

**Figure 2:**
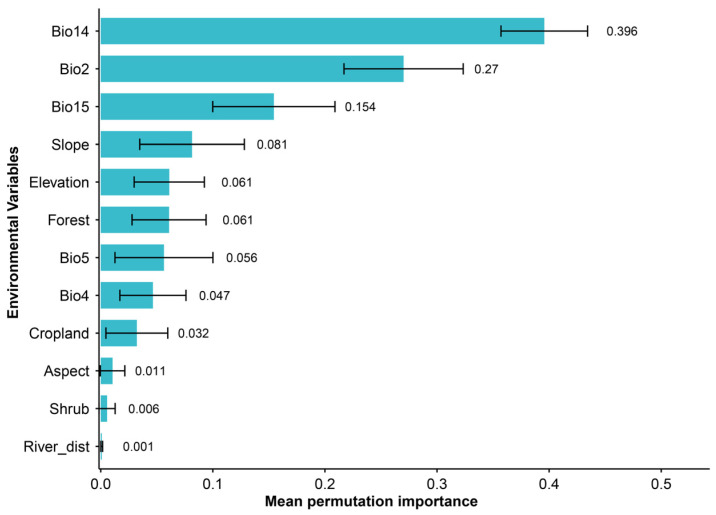
Mean permutation importance of the 12 selected environmental variables in predicting the potential distributions of François’ langur in the current period.

The distribution model for François’ langur demonstrated excellent predictive performance, with the area under the receiver operating characteristic curve (AUC) exceeding 0.99 and True Skill Statistic (TSS) values above 0.88. The relative contributions of environmental predictors revealed that both climatic and non-climatic variables made substantial contributions in shaping the distribution patterns of the target species. Among the climatic predictors, BIO14 and BIO2 were the most influential variables, followed by BIO15 (Figure 2). Land-use variables also contributed substantially to model performance; the proportion of forest cover emerged as the most important land-use predictor, whereas other land-use categories showed relatively minor effects. Topographic factors further improved model explanatory power; slope and elevation were identified as the key determinants of species distribution, while aspect had comparatively smaller contributions (Figure 2). The current distribution pattern is jointly shaped by climatic conditions, habitat structure, and topographic heterogeneity, highlighting the importance of integrating multiple environmental dimensions in François’ langur distribution modeling.

### 3.2. Analysis of Response Curve

**Figure 3:**
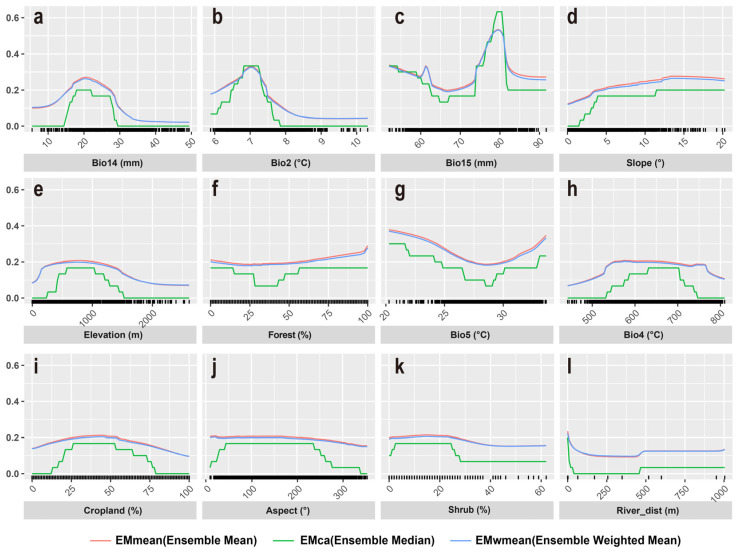
Response curves of 12 environmental variables in the ecological niche model for François’ langur. (**a**), Bio14; (**b**), Bio2; (**c**), Bio15; (**d**), Slope; (**e**), Elevation; (**f**), Forest (%); (**g**), Bio5; (**h**), Bio4; (**i**), Cropland; (**j**), Aspect; (**k**), Shrub; (**l**), River_dist.

Species response curves illustrated the relationships between environmental variables and the probability of François’ langur presence, reflecting the ecological tolerance and habitat preferences of François’ langur. Based on the response curves (Figure 3), occurrence probability peaked when precipitation of the driest month (BIO14) was approximately 20 mm (suitable range: 15–27 mm) and precipitation seasonality (BIO15) was around 85. Mean of monthly (max temp min temp) (BIO2) showed an optimum near 7 °C (Figure 3). Probability of presence increased with slope and was highest at elevations between 500 and 1000 m (Figure 3).

### 3.3. Current Potentially Suitable Habitats for François’ Langur

**Figure 4:**
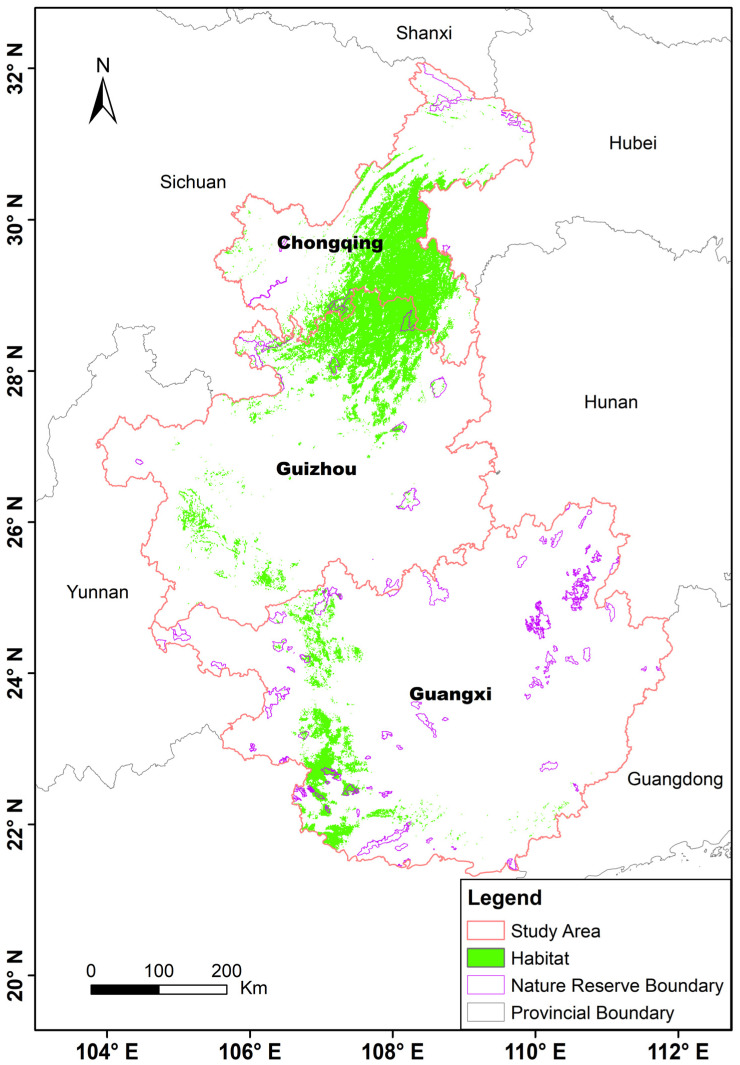
Predicted current potential distribution of François’ langur based on species occurrence records and environmental variables analyzed in this study.

Under current conditions, the total suitable habitat area was 53,109 km^2^, representing 10.75% of the total study area (494,176 km^2^) (Figure 4). Suitable habitats were mainly concentrated in southwestern China. Among the regions, Chongqing contained 23,208 km^2^ of suitable habitat, accounting for 43.36% of the total suitable habitat area, followed by Guizhou (20,265 km^2^; 38.16%) and Guangxi (9816 km^2^; 18.48%) (Table 1).

### 3.4. Dynamic Changes in the Suitable Habitats for François’ Langur Under Different Combinations of Climate Scenarios/Years

**Figure 5:**
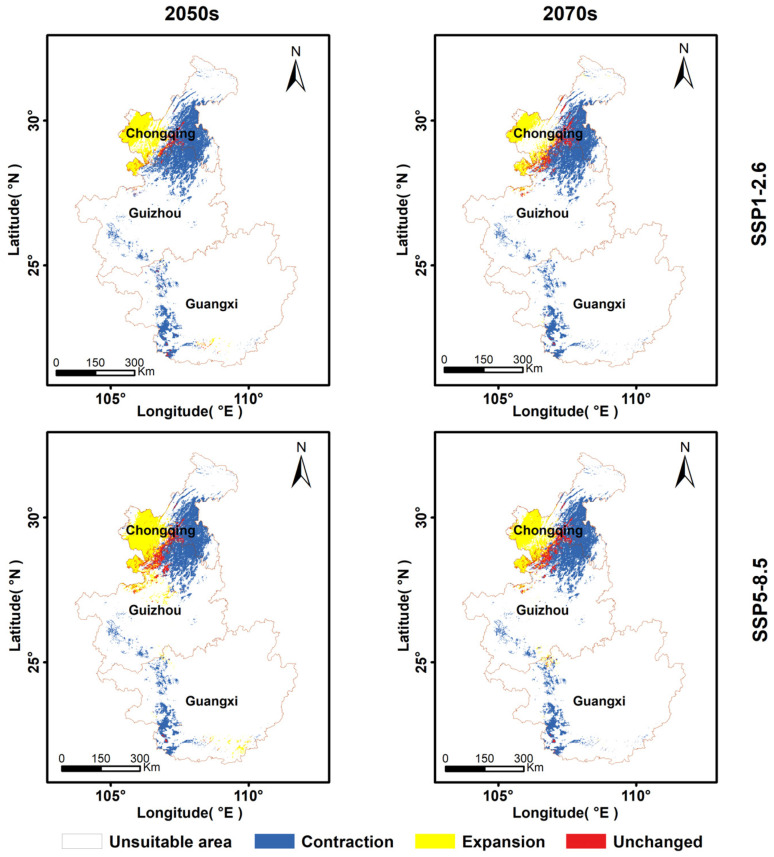
The predicted changes in suitable habitat of the François’ langur between current and different future scenarios by the COMB models: SSP1-2.6, SSP5-8.5 by the 2050s, and SSP1-2.6, SSP5-8.5 by the 2070s. Contraction: the gid cells that were predicted to be suitable under the current period but become unsuitable under the future period. Unchanged: the gid cells that were predicted to be suitable under both the current and future periods. Expansion: the gid cells that were predicted to be unsuitable under the current period but become suitable under the future period. Absence: the gid cells that were predicted to be unsuitable under both the current and future periods.

Under future climate and land-use scenarios, the spatial distribution of suitable habitat is projected to undergo substantial redistribution relative to the current conditions. Under the SSP1-2.6 scenario, suitable habitat is projected to decline to 20,303 km^2^ by the 2050s (Figure 5). Of this area, 3071 km^2^ remain unchanged (retention rate of 5.78%), 17,232 km^2^ are expansion areas. By the 2070s, the total suitable habitat slightly decreases to 19,581 km^2^, including 6185 km^2^ of Unchanged habitat (11.65%) and 13,396 km^2^ of expansion areas (Table 2).

Under the SSP5-8.5 scenario, habitat turnover remains pronounced. By the 2050s, total suitable habitat is projected to decline to 32,039 km^2^, with 6910 km^2^ remaining stable and 25,129 km^2^ expansion areas (Figure 5). By the 2070s, suitable habitat is projected to decline to 25,237 km^2^, including 6309 km^2^ of unchanged habitat (11.88%) and 18,928 km^2^ of expansion areas. Suitable habitat is projected to shift northward and toward higher elevations (Table 2). Guangxi shows the most pronounced reduction in suitable habitat, whereas Chongqing exhibits the greatest habitat expansion (Figure 5).

## 4. Discussion

### 4.1. Environmental Variable Predictors and Model Performance

The results indicate that the François’ langur distribution model achieved excellent predictive performance (AUC > 0.99; TSS > 0.88), suggesting high reliability in predicting suitable habitats. Climatic variables were the primary determinants of species distribution, with precipitation of the driest month (BIO14) and mean of monthly temperature (max temp–min temp) (BIO2) exerting the strongest influences, followed by precipitation seasonality (BIO15). Climate strongly affects vegetation productivity, water availability, and seasonal food supply, which in turn shape primate distributions [17,27,33,46]. Non-climatic variables also played important roles. Forest cover was the most influential land-use predictor, consistent with studies showing that François’ langurs depend heavily on forested habitats for feeding, resting, and protection [47,48,49,50]. Topographic factors further improved model performance, with slope and elevation emerging as key determinants. Steep slopes experience lower levels of human disturbance and can provide suitable sleeping and refuge sites, which may enhance habitat security for the species [48,51,52,53,54].

Under current conditions, suitable habitat covers 53,109 km^2^ (10.75% of the study area) and is concentrated in southwestern China, indicating a regionally clustered distribution. Response curves suggest that François’ langurs prefer moderately dry conditions, relatively stable precipitation regimes, and moderate daily temperature variability. Habitat suitability increased with slope and peaked at elevations between 500 and 1000 m, highlighting preferences for rugged, mid-elevation landscapes. These patterns reflect the François’ langur adaptation to karst mountain ecosystems and forested slopes, where complex terrain buffers climatic extremes and limits human disturbance [49,54,55,56]. The findings show that the current distribution of François’ langurs is shaped by the combined effects of climate, vegetation structure, and topographic heterogeneity, emphasizing the need to integrate multiple environmental factors when identifying conservation priorities.

### 4.2. The Current Potential Distribution of François’ Langur

Under current conditions, suitable habitat for François’ langur is mainly concentrated in southwestern China. Among the three key regions, Chongqing and Guizhou contain larger and more continuous areas of suitable habitat, whereas Guangxi supports comparatively smaller and more fragmented patches. This pattern likely reflects differences in habitat integrity and human disturbance. Mountainous forests in Chongqing and northern Guizhou remain relatively intact and provide extensive karst landscapes, forest cover, and steep terrain that offer secure sleeping sites and refuge from disturbance [48,49,54]. Although Guangxi historically supported substantial François’ langur populations, recent studies have documented sharp population declines associated with habitat loss, fragmentation, and human activities such as agriculture and infrastructure expansion [16,32,57,58]. The reduced extent and increased fragmentation of suitable habitat in Guangxi therefore likely reflect long-term habitat degradation and landscape isolation, which can limit dispersal and reduce population viability.

### 4.3. The Future Potential Distribution of François’ Langur

Under future climate and land-use scenarios (SSP1-2.6 and SSP5-8.5), the results indicate that suitable habitat for François’ langur will undergo substantial reorganization accompanied by an overall decline. By the 2050s and 2070s, both scenarios show pronounced habitat turnover: although some areas remain stable, losses and newly suitable areas occur simultaneously, reflecting continuous redistribution rather than simple contraction. Spatial analysis of the projected maps reveals a clear northward and upslope migration trend, as suitable habitats shift from the low-latitude karst regions of Guangxi toward higher-latitude mountainous areas. This pattern is primarily driven by the synergistic effects of increasing temperatures and limited dry-season precipitation (BIO14), which force the species to track cooler and more stable microclimates [6,59]. Guangxi is projected to experience the most severe habitat reduction, with formerly occupied areas further shrinking and becoming increasingly fragmented. In contrast, mountainous regions of Chongqing and northern Guizhou are expected to become future centers of suitability, with Chongqing showing notable potential expansion. This geographic shift suggests that conservation priorities should adjust accordingly, focusing on key refugial landscapes such as Mayanghe National Nature Reserve, Dashahe National Nature Reserve, and Jinfoshan National Nature Reserve, which are likely to function as core strongholds for sustaining future populations.

The projected redistribution aligns with the ecological characteristics of François’ langur, a specialized primate that depends on forest cover on steep limestone karst terrain and exhibits sensitivity to habitat disturbance and fragmentation [60,61]. Climate warming may alter vegetation structure, water availability, and seasonal resource patterns in karst ecosystems, thereby influencing food availability and shelter conditions. As temperatures rise, mid-elevation mountainous areas with complex topography may provide favorable microclimates and reduced human disturbance, enhancing their role as climate refugia. Similar climate-driven elevational shifts and range contractions have been reported for other primates and forest-dependent mammals [12,13,62,63]. Given the expected fragmentation and northward shift in suitable habitats, strengthening landscape connectivity will be essential. Establishing ecological corridors, restoring degraded forest patches, and promoting cross-regional conservation cooperation can enhance dispersal and gene flow, thereby improving the capacity of François’ langur to adapt to ongoing climate change and human pressures.

## 5. Conclusions

This study indicates that the suitable habitat of François’ langur is jointly determined by climatic conditions, forest cover, and topographic features, with steep terrain and mid-elevation environments providing favorable future conditions. At present, suitable habitat covers 53,109 km^2^ (10.75% of the study area) and is mainly concentrated in southwestern China, particularly in Chongqing and Guizhou. Under future climate and land-use scenarios (SSP1-2.6 and SSP5-8.5), suitable habitat is projected to undergo marked redistribution and an overall declining trend by the 2050s and 2070s. Habitat turnover will remain high, with coexistence of unchanged, expansion, and contraction areas, indicating continuous spatial reorganization. The distribution center is projected to shift northward and toward higher elevations, consistent with species responses to climate warming. Nevertheless, the predictions are subject to uncertainties related to species occurrence data, future land-use trajectories, and the exclusion of behavioral adaptation and fine-scale human disturbances. Long-term monitoring and adaptive management will therefore be necessary to refine conservation strategies and improve the future resilience of François’ langur populations.

## Figures and Tables

**Table 1:** Projected suitable habitat area (km^2^) and its regional distribution (%) for François’ langur under current (2020) and future climate scenarios (SSP1-2.6 and SSP5-8.5). Regional percentages were calculated as the proportion of total suitable habitat area occurring within Chongqing, Guizhou, and Guangxi.

Climate Scenario	Year	Total Area (km^2^)	Chongqing (km^2^)	Chongqing (%)	Guizhou (km^2^)	Guizhou (%)	Guangxi (km^2^)	Guangxi (%)
Current	2020	53,109	23,028	43.36	20,265	38.16	9816	18.48
SSP1-2.6	2050	20,303	16,707	82.29	2809	13.84	787	3.87
	2070	19,581	14,401	73.55	4980	25.43	200	1.02
SSP5-8.5	2050	32,039	21,151	66.02	9538	29.77	1350	4.21
	2070	25,237	18,475	73.21	6058	24.01	704	2.78

**Table 2:** Projected changes in suitable habitat area (km^2^) under future climate scenarios (SSP1-2.6 and SSP5-8.5) for François’ langur in 2050s and 2070s. Unchanged habitat denotes areas remaining suitable over time, whereas Contraction habitat indicates currently suitable areas projected to become unsuitable. Expansion habitat represents areas projected to become suitable in the future. Net change equals the future total suitable habitat minus the current total suitable habitat.

Habitat Change Category	2050s SSP1-2.6 (km^2^)	2050s SSP5-8.5 (km^2^)	2070s SSP1-2.6 (km^2^)	2070s SSP5-8.5 (km^2^)
Unchanged habitat	3072	6954	6185	6310
Contraction habitat	50,153	46,271	47,040	46,915
Expansion habitat	17,235	25,294	13,396	18,929
Total habitat (future)	20,307	32,248	19,581	25,239
Total habitat (current)	53,109	53,109	53,109	53,109
Net change	−32,918	−20,977	−33,644	−27,986

## Data Availability

The original contributions presented in the study are included in the article. Further inquiries can be directed to the corresponding authors.
